# Investigating the Efficacy of Zinc and Vitamin A in Treating Pediatric Community-Acquired Pneumonia

**DOI:** 10.7759/cureus.52197

**Published:** 2024-01-13

**Authors:** Aqsa Atta, Ayesha Aftab, Ayesha Shafqat, Muhammad Hamza Yousuf, Akbar Ahmed, Hannah Pirzada, Humna Khalid, Natasha E Hastings

**Affiliations:** 1 Human Nutrition and Dietetics, Nishtar Medical University, Multan, PAK; 2 Pharmacology, Al Nafees Medical College and Hospital, Islamabad, PAK; 3 Physiology, HBS (Hazrat Bari Imam Sarkar) Medical College, Islamabad, PAK; 4 Emergency Department, Imam Clinic, Karachi, PAK; 5 Gynaecology and Obstetrics, Ziauddin University Hospital, Karachi, PAK; 6 Pharmacology, Bakhtawar Amin Medical College, Multan, PAK; 7 Internal Medicine/Dermatology, Bahawal Victoria Hospital, Bahawalpur, PAK; 8 School of Medicine, St. George's University, St. George's, GRD

**Keywords:** supplement, vitamin a, zinc, community-acquired pneumonia, pneumonia, pediatric

## Abstract

Background: Community-acquired pneumonia (CAP) poses a significant global health challenge, even more so for children less than five years old. Nutritional interventions, such as zinc and vitamin A supplementation, are gaining attention for their therapeutic potential in enhancing recovery and minimizing pneumonia severity in pediatric patients.

Objective: To assess the therapeutic benefits of zinc and vitamin A supplementation in pediatric CAP patients under five years old and to advocate for their use in clinical settings.

Methodology: Three groups were formed in a randomized controlled trial conducted from October 2022 to September 2023, to address zinc and vitamin A supplementation in pediatric patients under five years old in the intensive care unit with severe pneumonia. Group 1 received zinc supplementation, group 2 received vitamin A supplementation, and group 3 served as the control group, receiving antibiotic treatment exclusively for pneumonia. This treatment comprised either a β-lactam (amoxicillin-clavulanate, commonly referred to as Augmentin) administered orally at 500 mg/125 mg three times a day, Augmentin 875 mg/125 mg orally twice daily, or Augmentin 2000 mg/125 mg orally once daily. Additionally, the control group received a macrolide (azithromycin or clarithromycin) or doxycycline at a dosage of 100 mg orally twice daily.* *Linear regression analysis identified statistically significant decreases in both length of hospital stay and active pneumonic effusion.

Results: The study encompassed 90 pediatric pneumonia patients with an age range of six to 55 months. Multiple linear regression analysis showed that both vitamin A and zinc led to a significant decrease in hospitalization length by 2.39 days (p < 0.01, 95% CI: 4.19-0.47) and 3.17 days (p < 0.01, 95% CI: 5.19-1.31), respectively. In comparison to the control group, both the vitamin A and zinc supplementation groups were linked to a shorter pneumonic effusion duration (p < 0.001).

Conclusion: Both interventions significantly reduced the duration of hospitalization (2.39 days for vitamin A and 3.17 days for zinc) and pneumonic effusion compared to the control group. These findings highlight the potential of zinc and vitamin A as valuable additions to standard CAP treatment regimens, potentially leading to improved clinical outcomes and reduced healthcare burdens.

## Introduction

Community-acquired pneumonia (CAP) continues to be a major worldwide health problem, especially for children under five years old [1]. This respiratory illness has been a major source of morbidity and death for children in this age group, placing a significant strain on healthcare systems [2]. The potential use of nutritional treatments as additional therapy to improve recovery and lessen the severity of pneumonia in pediatric patients has drawn interest. Zinc and vitamin A are two micronutrients that have come to light as viable options with immunomodulatory qualities that may influence the development of pediatric CAP [3,4].

Zinc is essential for a healthy immune system. Zinc is known to be associated with two immunological processes: immunological cell development and physiology. More specifically, zinc is responsible for regulating intracellular signaling pathways in innate and adaptive immune cells. Patients with zinc deficiencies often present with lymphopenic symptoms, e.g., decreased T-helper (Th) to cytotoxic T-cell ratios, decreased natural killer (NK) cell activity, and increased monocyte cytotoxicity [4,5]. These immunomodulatory functions encouraged our selection of zinc as a viable therapeutic agent in the treatment of CAP.

Vitamin A is important to maintain immune responses to infections and retain the integrity of mucosal surfaces. This micronutrient plays a role in the production of inflammatory and immunomodulatory cytokines as well as reducing reactive oxygen species (ROS), and its lack thereof predisposes patients to infectious diseases, such as CAP. Vitamin A deficiency is common in children and accounts for 2% of all deaths of those under five years old [6].

Both micronutrients, i.e., zinc and vitamin A, are essential to the body's defense systems, and shortages in either one may make it more difficult for the immune system to successfully fight infections [7,8]. Few studies have directly examined the treatment benefits of these micronutrients specifically in the setting of pediatric CAP, despite the fact that both nutrients have proven beneficial functions in immune function [4,9,10]. By identifying the effects of zinc and vitamin A on the rate of recovery and pneumonic effusion of children suffering from CAP under the age of five, this research seeks to close this gap.

This study intends to provide important insights into the possible advantages of zinc and vitamin A supplementation by carrying out a thorough analysis and assisting in the development of evidence-based therapies for enhancing outcomes in pediatric CAP patients. We hope that further investigation into this research will reveal subtleties that will help medical practitioners make well-informed judgments about whether or not to include certain micronutrient therapies in pediatric CAP management regimens. The results of this study could open the door to more focused and successful approaches to lessening the negative effects of CAP on the health and well-being of young children in this age group.

This study's main goal was to evaluate and examine the therapeutic benefits of zinc and vitamin A supplementation in children under the age of five years with CAP.

## Materials and methods

Study design

**Figure 1:**
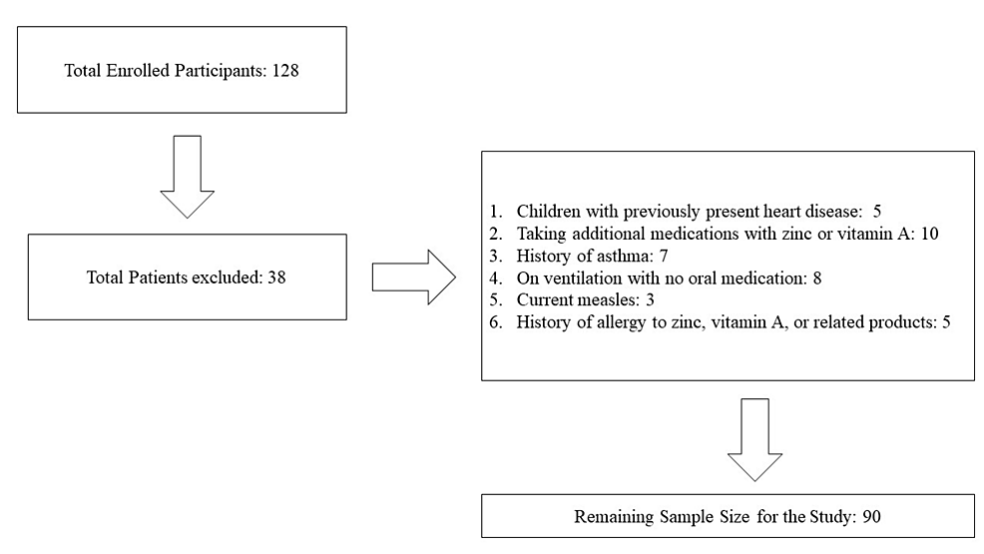
Participant selection flowchart.

A multi-center, randomized controlled trial was conducted from October 2022 to September 2023 at three distinguished healthcare institutions in Pakistan: Nishtar Hospital, Multan; Aga Khan University Hospital, Karachi; and Al Nafees Medical College and Hospital, Islamabad. This study aimed to investigate the effectiveness of different treatment strategies for pediatric patients less than five years old with severe pneumonia in both pediatric and gynecology departments. Among the 128 initially enrolled participants, 38 were excluded for various reasons, including five children with pre-existing heart disease, 10 on additional medications containing zinc or vitamin A, seven with a history of asthma, eight on ventilation with no oral medication, three with current measles, and five with a history of allergy to zinc, vitamin A, or related products (Figure 1). As a result, the study's remaining sample size was 90 participants. Eligible patients were randomly assigned into three distinct groups to receive different treatment interventions.

Inclusion criteria

Pneumonia-diagnosed pediatric intensive care unit patients (n = 90) under the age of five years were included in the study.

Exclusion criteria

Children under five years old with previously present heart disease, those taking medications in addition to zinc supplementation or vitamin A, children who showed signs and symptoms of asthma, those on ventilation with no oral medication, those with active measles at the time, and those with a history of allergy to zinc, vitamin A, or products containing these ingredients were not included in the study.

Pneumonia treatment: zinc, vitamin A, and control groups

Group 1, referred to as the zinc group, received zinc sulfate syrup (10 mg/5 ml) for a duration of seven days. Infants aged below one year were administered 10 mg orally once daily, while children aged one to five years received 20 mg orally once a day. In group 2, known as the vitamin A group, patients received soft gelatin capsules containing 50,000 IU/1.25 mg of vitamin A orally on days one and five for infants under one year and 100,000 IU on days one and five for children between one and five years old. Group 3 served as the control group, receiving antibiotic treatment exclusively for pneumonia. This treatment comprised either a β-lactam (amoxicillin-clavulanate, commonly referred to as Augmentin) administered orally at 500 mg/125 mg three times a day, Augmentin 875 mg/125 mg orally twice daily, or Augmentin 2000 mg/125 mg orally once daily. Additionally, the control group received a macrolide (azithromycin or clarithromycin) or doxycycline at a dosage of 100 mg orally twice daily (Table 1).

**Table 1:** Summary of treatment protocols for pediatric patients in severe pneumonia study.

Group	Treatment	Dosage	Duration
Group 1 (zinc)	Zinc sulfate syrup	10 mg/5 ml	7 days
		Under 1 year: 10 mg orally once a day	
		1-5 years: 20 mg orally once a day	
Group 2 (vitamin A)	Soft gelatin capsules	50,000 IU/1.25 mg of vitamin A	Days 1 and 5
		Under 1 year: 50,000 IU orally on both days	
		1-5 years: 100,000 IU orally on both days	
Group 3	Control group	Antibiotic treatment for pneumonia	-
		β-lactam (amoxicillin-clavulanate)	
		500 mg/125 mg orally three times a day	
		875 mg/125 mg orally twice daily	
		2000 mg/125 mg orally once daily	
		Macrolide (azithromycin or clarithromycin)	
		Doxycycline 100 mg orally twice daily	

Patient monitoring during supplementation

Every day, the doctor performed medical checks, and the nurses used pulse oximetry to track oxygen saturation, respiration rate, and body temperature. A side effect monitoring form was used to carefully follow the adverse effects of zinc supplementation, which included nausea, vomiting, diarrhea, tarry stool, and stomach discomfort, and vitamin A supplementation, which included nausea, vomiting, diarrhea, liver damage, and skin irritation. Any incidence of the listed adverse effects was recorded, and the clinical approach to managing them was noted.

Statistical analysis

Statistical analyses were conducted using SPSS version 27 (IBM Corp., Armonk, NY). The Shapiro-Wilk test was employed to assess the normality of continuous data. Data that exhibited normal distribution were presented as mean ± standard deviation (SD), while skewed data were presented as median and range. Comparisons for normally distributed data were performed using the Student's t-test, while comparisons for skewed data were made using the Mann-Whitney test. Additionally, the chi-square test was applied for categorical variables. Statistical significance was established at a two-sided p-value of less than 0.05.

Ethical statement

This study was approved by the Institutional Review Board (IRB) of Nishtar Medical University, Multan, with approval number N2022-0098-1.

## Results

The trial was successfully completed by 90 patients, with no dropouts. The individuals' ages ranged from six to 55 months old, with a median age of 28 months. At first, it was discovered that the baseline data on gender, age, grade of pneumonia, actual body weight, and the presence or absence of pneumonic effusion were all similar. The individuals' initial demographic features are shown in Table 2. Age is presented as a median and range, hinting at a non-normal distribution (ANOVA, p = 0.99). Interestingly, the age distribution is quite similar across groups, though the zinc group leans slightly younger. Gender distribution leans slightly toward females with a 63.33% majority (chi-squared, p = 0.42). Considering weight, both the mean and standard deviation for each group suggest a normal distribution. The vitamin A group seems to have a slightly lower average weight than the others (Kruskal-Wallis, p = 0.36). Body weight categories (underweight, healthy, overweight, obese) also reveal a comparable distribution across groups, with no significant differences observed (chi-squared, p = 0.78). Pneumonia severity was categorized into grades 3 and 4. The distribution of these grades is nearly identical across groups, meaning participants started with similar disease severity (chi-squared, p = 1.00). Similarly, the presence or absence of pneumonic effusion (fluid in the lungs) follows a comparable pattern across groups, with a slight majority (68.89%) not having effusion at the outset (chi-squared, p = 1.00).

**Table 2:** Comparative analysis of demographic and health parameters across control, vitamin A, and zinc groups in a study of 90 subjects. ^a ^Analysis of variance linear model (ANOVA). ^b ^Pearson’s chi-squared test. ^c ^Kruskal-Wallis rank-sum test.

Parameter	Control (n = 30)	Vitamin A (n = 30)	Zinc (n = 30)	Total (n = 90)	p-value
Age (months)					0.99^a^
Median (range)	32.0 (6.0-55.0)	27.0 (6.0-52.0)	25.0 (6.0-54.0)	28.0 (6.0-55.0)	
Infant	2 (6.67)	5 (16.67)	4 (13.33)	11 (12.22)	
Toddler	13 (43.33%)	16 (53.33%)	14 (46.67%)	43 (47.78%)	
Preschool	15 (50.0%)	9 (30.0%)	12 (40.0%)	36 (40.0%)	
Gender					0.42^b^
Female	17 (56.67%)	19 (63.33%)	21 (70.0%)	57 (63.33%)	
Male	13 (43.33%)	11 (36.66%)	9 (30.0%)	33 (36.67%)	
Actual body weight (kg)					0.36^c^
Mean (SD)	14.7 (3.2)	12.6 (3.6)	13.5 (3.4)	13.6 (3.4)	
Underweight	2 (6.67%)	5 (16.67%)	3 (10.0%)	10 (11.11%)	
Healthy	13 (43.33%)	14 (46.66%)	12 (40.0%)	39 (43.34%)	
Overweight	4 (13.33%)	2 (6.67.0%)	5 (16.67%)	11 (12.22%)	
Obese	11 (36.67.0%)	9 (30.0%)	10 (33.33%)	30 (33.33%)	
Severity of pneumonia					1.00^b^
Grade 3	14 (46.67%)	12 (40.0%)	13 (43.33%)	39 (43.33%)	
Grade 4	16 (53.33%)	18 (60.0%)	17 (56.67%)	51 (56.67%)	
Effusion					1.00^b^
Absent	18 (60.0%)	21 (70.0%)	23 (76.67%)	62 (68.89%)	
Present	12 (40.0%)	9 (30.0%)	7 (23.33%)	28 (31.11%)	

The ANOVA statistical evaluation of the data revealed a significant decrease in the duration of pneumonic effusion for both the zinc and vitamin A groups compared to the control group, with a statistically significant p-value of less than 0.001 (Table 3).

**Table 3:** The timeframe of pneumonic effusion across investigated groups.

Term	Degrees of freedom	Sum of squared errors	Mean squared errors	F-statistic	p-value
Treatment	4	131	65.9	17.6	<0.001
Residuals	18	63.4	3.98		

Delving deeper into the effects of zinc and vitamin A on effusion duration, a post-hoc analysis using the non-parametric Mann-Whitney U test revealed encouraging results for both groups compared to the control (Table 4). Zinc took the lead, with a statistically significant mean reduction of 6.29 days in effusion duration (p < 0.01), outperforming control in clearing lung fluid. Vitamin A followed close behind with a 3.45-day reduction (p = 0.02), solidifying its role in effusion resolution. While zinc showed a slight edge with a 2.83-day advantage. This difference did not reach conventional levels of statistical significance as a direct comparison through Mann-Whitney U yielded an insignificant p-value (p = 0.07).

**Table 4:** Post-hoc analysis: contrasting pneumonic effusion across studied groups.

Contrast	x̄1	x̄2	x̄1-x̄2	Lower bound	Upper bound	p-value
				95% confidence interval		
Vitamin A - control	8.83 (±0.75)	12.30 (±2.75)	-3.47	-6.28	-0.62	0.02
Zinc - control	6.00 (±1.67)	12.29 (±2.75)	-6.29	-9.12	-3.45	<0.01
Zinc - vitamin A	6.00 (±1.67)	8.83 (±0.75)	-2.83	-5.77	0.11	0.07

The “Effusion-treatment” and “Grade-treatment” interactions (p = 0.86 and 0.97) revealed that the response to supplementation (reduced hospitalization duration) remained independent of both effusion presence and pneumonia severity (grades 3 and 4; Table 5). This suggests that vitamin A and zinc offered comparable benefits regardless of effusion or disease severity, potentially broadening their application range. Table 4 also shows that the main effects emerged for “Effusion” (p < 0.001) and “Pneumonia Grade” (p < 0.001), indicating that participants with effusion and higher pneumonia grades generally stayed longer, highlighting their roles in influencing illness severity and recovery time. A significant “Treatment” main effect (p < 0.01) confirmed that both vitamin A and zinc effectively reduced hospitalization duration compared to control, solidifying their overall benefits in shortening hospital stays for children with CAP.

**Table 5:** Examining hospitalization duration: impact of pneumonia grade and effusion presence in studied groups.

Term	Degrees of freedom	Sum of squared errors	Mean squared errors	F statistic	p-value
Effusion	1	560	560	49.3	<0.001
Treatment	2	149	74.3	6.54	<0.01
Effusion-treatment	2	3.52	1.76	0.16	0.86
Residuals	69	784	11.4		
Pneumonia grade	1	497	497	40.4	<0.001
Treatment	2	149	74.3	6.03	<0.01
Grade-treatment	2	0.35	0.18	0.01	0.97
Residuals	69	850	12.3		

A multiple linear regression analysis (Table 6) revealed that both vitamin A and zinc supplementation significantly reduced hospitalization duration for children with pneumonia. Vitamin A was associated with a marginally significant reduction of 2.39 days (p = 0.01, 95% CI: 4.19-0.47), while zinc supplementation yielded a more pronounced effect, averaging a 3.17-day reduction (p < 0.01, 95% CI: 5.19-1.31). These findings suggest that both micronutrients hold promise in facilitating faster recovery and shorter hospital stays for children with CAP.

**Table 6:** Multiple linear regression analysis of factors influencing hospitalization duration.

Variables		Estimates	95% CI	p-value
Gender	Male	0.26	-1.18-1.68	0.73
	Female			
Age	Infant	-0.01	-1.16-2.31	0.46
	Toddler	-0.04	-2.62-2.69	
	Preschool	1.08	-1.79-3.84	
Actual body weight (kg)	Underweight	0.64	-2.28-3.63	0.67
	Healthy	0.52	-1.71-2.84	0.58
	Overweight	0.52	-1.71-2.90	0.63
	Obese	0.07	-1.64-1.80	0.93
Effusion	Present	3.98	1.86-6.21	0.01
	Absent			
Pneumonia grade	Grade 3 and grade 4	3.61	1.42-4.80	0.01
Treatment	Zinc	-3.17	-5.19-1.31	0.01
	Vitamin A	2.39	-4.19-0.47	0.01

## Discussion

Micronutrients like zinc and vitamin A are involved in how the immune system works and the healing process after infections. Studies have shown that children from low- and middle-income countries often have lower concentrations of these nutrients than what is necessary [10-12]. Zinc is necessary for immune system function and the metabolism of cells. Its absence results in a malfunction in the lung's epithelium-cell fusion [13,14]. Zinc is known to be useful for the medical management of respiratory tract infections because it inhibits the enzyme angiotensin-converting enzyme 2 (ACE2), which in turn hinders the progression to become infected and shortens the duration of manifestations as well as the seriousness of the illness [15].

Vitamin A has been extensively researched for its impact on the immune system and is known as an anti-infectious vitamin because of its ability to regulate human immunological function [9,10]. Frequent respiratory tract infections have been linked to vitamin A deficiency. This is because vitamin A is one of the fat-soluble vitamins that greatly affects immune system health, and long-term vitamin A deficiency raises rates of illness and death, particularly in the pediatric population [16].

A cross-sectional investigation revealed that vitamin A intervention reduced the length of hospital stay and fever [17]. Regarding hospital stays, we discovered that the zinc or vitamin A treatments decreased the average number of days spent in the hospital by 3.17 days (95% CI: 5.19-1.31, p < 0.01) and 2.39 days (95% CI: 4.19-0.47, p = 0.01), respectively. This result is comparable to that of the studies by Valavi et al., who administered zinc at a dose of 2 mg/kg/day (maximum daily dose: 20 mg) for five days to children aged three to 60 months [18], and Reyes et al., who administered zinc at a dose of 15 mg twice daily until hospital discharge or for a maximum of seven days to children aged two to 60 months [19]. Nevertheless, Shah et al. discovered that giving children aged two to 60 months 10 mg of zinc twice a day for seven days had no appreciable impact on hospital stays among pneumonia patients [20].

In the current study, it was discovered that the zinc and vitamin A groups' effusion duration was noticeably shorter than that of the control group. When the zinc group was compared to the vitamin A group, it was shown that zinc produces a non-significant three-day decrease in the effusion's length. The exact processes by which zinc and vitamin A shorten hospital stays or reduce pleural effusions are unclear, although they may be related to their antimicrobial, anti-inflammatory, and tissue growth control effects [4]. Despite Shah et al.'s study recording 42% and 35.35%, respectively, for the male and female patients' susceptibilities to pneumonia infection, we found that the female patients' susceptibility was higher (63.33%) [20]. There might be variations in research populations, geographical locations, time periods, methodology, and sample sizes contributing to the disparity in pneumonia susceptibility.

Limitations of the study

Limited sample size, short follow-up period, and narrow inclusion criteria preclude definitive conclusions about zinc and vitamin A supplementation for pediatric CAP. Further research with larger sample sizes, longer follow-up periods, and broader inclusion criteria is needed to confirm these findings and establish definitive guidelines for their use in clinical practice.

## Conclusions

Zinc and vitamin A supplements have the ability to significantly improve pediatric CAP symptoms, evident in reductions in the duration of hospitalization and pneumonic effusion. Both micronutrients significantly shorten the duration of pneumonic effusion and this study advocates for clinical implementation of both, with zinc having a particularly notable impact. Regardless of demographic characteristics, multiple linear regression analysis reveals their contributions to a shorter hospital stay and more efficient pneumonic effusion resolution. These results represent a significant advancement in focused therapies to improve recovery outcomes for children with CAP and provide a strong basis for evidence-based strategies.
